# Acceptance of Transportation Technologies by Aging Adults

**DOI:** 10.1093/geroni/igaa057.1822

**Published:** 2020-12-16

**Authors:** Neil Charness, Dustin Souders, Ryan Best, Nelson Roque, JongSung Yoon, Cary Stothart

**Affiliations:** 1 Florida State University, Tallahassee, Florida, United States; 2 Purdue University, West Lafayette, Indiana, United States; 3 Rand Corporation, Santa Monica, California, United States; 4 Pennsylvania State University, University Park, Pennsylvania, United States; 5 University of South Dakota, Vermillion, South Dakota, United States; 6 U.S. Army Research Institute for the Behavioral and Social Sciences., Fort Leavenworth, Kansas, United States

## Abstract

Older adults are at greater risk of death and serious injury in transportation crashes which have been increasing in older adult cohorts relative to younger cohorts. Can technology provide a safer road environment? Even if technology can mitigate crash risk, is it acceptable to older road users? We outline the results from several studies that tested 1) whether advanced driver assistance systems (ADAS) can improve older adult driving performance, 2) older adults’ acceptance of ADAS and Autonomous Vehicle (AV) systems, and 3) perceptions of value for ADAS systems, particularly for blind-spot detection systems. We found that collision avoidance warning systems improved older adult simulator driving performance, but not lane departure warning systems. In a young to middle-aged sample the factor “concern with AV” showed age effects with older drivers less favorable. Older drivers, however, valued an active blind spot detection system more than younger drivers.

